# Biology, taxonomy, genetics, and management of *Zymoseptoria tritici*: the causal agent of wheat leaf blotch

**DOI:** 10.1080/21501203.2023.2241492

**Published:** 2023-08-08

**Authors:** Girma Ababa

**Affiliations:** Department of Plant Protection (Plant Pathology), Holetta Agricultural Research Center (HARC), Ethiopian Institute of Agricultural Research (EIAR), Holetta, Addis Ababa, Ethiopia

**Keywords:** Biology, classification, control methods, variability, wheat, *Zymoseptoria tritici*

## Abstract

*Septoria tritici* blotch or *Septoria* leaf blotch has been used for long time, but leaf blotch is a correct disease name. Moreover, *Lb* resistant gene is the correct name, but, not *Stb* gene. It has sexual and asexual parts on the mycelia, known as heterothallic fungi. Its pathogenic diversity ranged from 40% to 93% and has produced a wide variety of *AvrLb6* haplotypes. *M. graminicola* has a plasmogamy and karyogamy sexual process. The pathogen can use macropycnidiospores, micropycnidiospores, and pycnidia vegetative growths for infection and overwintering. Synthetic M3, Kavkaz-K4500, Synthetic 6×, and TE9111 wheat genotypes have horizontal resistance. Avirulence (Avr) genes in *Z. tritici* and their matching wheat (R) genes indicate gene for gene mechanisms of resistance. Twenty-two R genes (vertical resistance) have been identified. In both horizontal and vertical resistance, different *Lb* genes have been broken down due to new *Z.tritici* virulent gene and currently *Lb19* resistant gene is being recommended. Mixing of resistant and susceptible cultivars is also the most effective management strategy. Moreover, different cultural practices and biological control have been proposed. Lastly, different fungicides are also available. However, in developing countries cultivar mixture, isolates diversity, biological control, and epidemic studies have been greatly missed.

## 1. Introduction

Wheat is a highly productive crop and produced all over the world, but the production of this crop is hindered by different biotic and abiotic factors. Leaf blotch disease is one of the most important fungal diseases. Quaedvlieg et al. (2004) identified *Septoria tritici* as the causal agent of leaf blotch disease, but, later advocated *Zymoseptoria tritici* (teleomorph: *Mycosphaerella graminicola*) (Quaedvlieg et al. 2011). *Z. tritici* is an apoplastic pathogen species with a hemibiotrophic life cycle (Ponomarenko et al. 2011; Fones and Gurr 2015; Savary et al. 2019).

Small chlorotic patches on the foliage are the first signs of leaf blotch disease. The lesions turn light tan as they grow and produce fruiting bodies that are deeper in colour. Especially on seedlings or leaves that were young when infected, lesions on mature leaves are frequently long, narrow, and defined by leaf veins, but they can also be shaped irregularly or be oval (Ponomarenko et al. 2011).

Leaf blotch disease is currently a significant and ongoing threat to wheat growers worldwide (Zhan et al. 2003; Ponomarenko et al. 2011). In fields with wheat cultivars susceptible to leaf blotch disease during severe epidemics, yield losses were 30% to 54% (Ponomarenko et al. 2011), 50.1% (Berraies et al. 2014). Furthermore, important wheat production areas in Ethiopia had a 25% to 82% loss in wheat output as a result of the frequency and intensity of leaf blotch disease infestations (Bekele 1985). However, another report showed a 41% yield loss in grain production (Takele et al. 2015).

Leaf blotch disease is widely distributed around the world, including in the largest wheat-producing countries of Argentina, Ethiopia, the United States, the Netherlands, Russia, New Zealand, Iran, Tunisia, Morocco, and Australia (Ponomarenko et al. 2011). The sexual reproduction of the pathogen regulates both pathogen populations and the quantity of primary inoculum (wind-dispersed ascospores) (Suffert et al. 2011). Hosseinnezhad et al. (2014) discovered that Iranian *Z. tritici* isolates showed significantly different virulence patterns, with virulence spectra ranging from 40% to 90% of the tested genotypes. Eight Iranian *Z. tritici* isolates were tested on 45 durum wheat landraces and the isolates’ pathogenicity spectrum ranged from 42% to 82% (Ghaneie et al. 2012). In Ethiopia, Mekonnen et al. (2020) found *Z. tritici* to have broad-spectrum virulence, successfully infecting 71% to 93% of the differential lines. In another country, Burcu et al. (2022) identified 28 pathotypes from 60 isolates in four provinces of the central Anatolia region of Turkey.

Regarding its significant importance and wide distribution where the wheat is cultivated, various management approaches have been investigated to control this disease. The most effective techniques for reducing *Z. tritici* infections are to grow mixed cultivars. The traditional method of leaf blotch disease protection has been the extensive application of fungicides supported by the use of a few resistant genes. However, the reproduction cycle of *Z. tritici* may contribute to the high genetic diversity of the pathogen, which could hasten the fungicides rapid loss of efficacy. There are currently resistant strains for each significant fungicide group used to combat them (Stammler and Semar 2011; Hillocks 2012; Van den Berg et al. 2013).

Wheat resistance at adult growth stage has been promoted for management of leaf blotch disease. Studies on the gene *Lb16q* revealed that it encodes a cysteine-rich receptor-like kinase that confers resistance of wheat at this growth stage (Zhong et al. 2017; Kema et al. 2018; Saintenac et al. 2018). The resistance of wheat crop to leaf blotch disease has been investigated using quantitative genetic resistance. According to genome-wide association study (GWAS), one or more chromosomes (1A, 1B, 1D, 2B, 3B, 4A, 5A, 6A, and 6B) may be associated with dult plant resistance (Muqaddasi et al. 2019; Alemu et al. 2021). Therefore, EBW174, Lorikeet, Synthetic M3, Kavkaz-K4500, Synthetic 6×, TE9111, Selam, Mangudo, EBW174, Borstvete, Ankar, Hereford, and Aring resistant materials have been suggested (Mekonnen et al. 2021, Alemu et al. 2021; Tidd et al. 2023). The resistance of durum and bread wheat crop in Ethiopia has been examined; therefore, Ababa et al. (2022) suggested Danda’a, HONQOLO, Digalu, Dashen, EJERSA, Alemtena, Mosobo, Hitosa, Robe, and Lelisso wheat cultivars showed resistant reaction to more than two isolates and considrered as horizontal resistance.

Moreover, for specific interactions between wheat cultivars and *Z. tritici* isolates, 22 *Lb* genes have been observed (Brading et al. 2002; Goodwin 2007; Ghaffary 2011; Orton et al. 2011; Brown et al. 2015). The gene *Lb6* encodes wall-associated kinases that confer gene–gene mechanisms of resistance (Saintenac et al. 2021). Consequently, scholars reported gene for gene or specific resistance found in KK variety, Synthetic 6×, SO852, Arina, TE 9111, Veranopolis, Olaf, and Shafir wheat genotypes (Eyal et al. 1985; Arraiano et al. 2001; Adhikari et al. 2004; Chartrain et al. 2004). However, the broad-spectrum resistance (*Lb16q*) gene transferred to wheat cultivars has been broken down (Dalvand et al. 2018; Kildea et al. 2020; Orellana-Torrejon et al. 2022). Moreover, the race-specific resistance (*Lb4*) gene is overcome (Jackson et al. 2000).

Although it is a debatable idea whether cultural practices can control leaf blotch disease, its severity was decreased as the ploughing frequency increased (Bailey et al. 2001; Gilbert and Woods 2001; Bankina et al. 2014; Fernandez et al. 2016; Ababa et al. 2021). Crop rotation is essential to prevent the sowing of wheat in paddocks with high levels of stubble-borne inoculum (Eyal and Levy 1987; Ponomarenko et al. 2011).

Despite the lack of information on the use of microbes as leaf blotch disease control agents, some of them have been published, such as *Paecilomyces lilacinus*, *Nigrospora sphaerica*, *Cryptococcus* sp., *Bacillus* sp., and *Bacillus* (Perello et al. 2002; Kildea et al. 2008). As a last option, in the absence of high-resistance wheat cultivars and significant disease pressure, fungicides are the primary means of disease control (Fraaije et al. 2012). A variety of management techniques, including crop rotation, resistant cultivars, biocontrol, the use of fungicides, and appropriate fertilisation can be utilised to reduce disease in conservation tillage systems (Bockus and Shroyer 1998; Krupinsky et al. 2002, 2004, 2007).

Different management strategies have been developed for this disease; nevertheless, the research on the most efficient time to apply fungicides has been mostly overlooked in developing countries. As a result, farmers have applied fungicides without being aware of the best time. Cultivar mixes are also one of the most important control method, but this is also underutilised in various developing countries. Different scholars have used “*Septoria tritici* blotch” as a disease name and *Z. tritici* (former *S. tritici*) as a causal agent (Makhdoomi et al. 2015; Teferi and Gebreslassie 2015; McDonald and Mundt 2016). However, the correct name for this disease is leaf blotch disease, and its causal agent is *Z. tritici* (Lillemo et al. 2011; Salgado and Paul 2016; Scala et al. 2020). *Stb* name was given for resistant gene in wheat based on *Septoria tritici* blotch disease. Moreover, the disease and R gene names and causal agent are not clearly used in different reports. Several *Lb* genes have been frequently broken down by new virulent isolates, but the status of the new virulent isolates is not available in different countries. Therefore, this review was conducted to state the correct disease name and causal agent, to demonstrate new virulent isolates, and to assess the biology of leaf blotch disease and control techniques.

## 2. Wheat blotch (leaf and glume blotch)

### 2.1. Causal agent of wheat blotch (leaf and glume blotch)

Most plant diseases are named after their symptoms (grey mould, downy mildew, Powdery mildew, ring rot, white silk, sclerotinia, etc.), characteristics (soft rot, wilt, damping off, scab, leaf blight, scab, bud blight, black rot, etc.), or pathogens (Pythium, Anthrax virus disease, etc.). Wheat blotch has been named in different ways for a long time. The disease was termed as “Septoria leaf blotch” (Makhdoomi et al. 2015; Teferi and Gebreslassie 2015; McDonald and Mundt 2016, 2016), “*Septoria tritici* blotch” (Figueroa et al. 2018). In these cases, “*Septoria tritici* blotch” or “Septoria leaf blotch” has been used as a disease name. However, *Septoria* represents a genus of plant pathogenic fungi and is extremely large in number (Quaedvlieg et al. 2004). Moreover, fungal species belonging to *Septoria* are among the most widespread leaf-spotting fungi worldwide. Therefore, according to these scientists, *Septoria tritici* is the causal agent of leaf blotch disease (Quaedvlieg et al. 2004). Later, *Septoria* was reclassified by the genus *Zymoseptoria* (Quaedvlieg et al. 2011). Leaf blotch disease has been used as a disease name (Lillemo et al. 2011; Salgado and Paul 2016; Scala et al. 2020). These indicate that different scholars have used leaf blotch name in different ways.

Accordingly, *Stb* name was given for the resistant gene in wheat based on the *Septoria tritici* blotch disease and has been used for a long time (Chartrain et al. 2004; Arraiano et al. 2009; Stephens et al. 2021). However, since leaf blotch disease is the correct disease name, *Lb* is also the correct name for resistant gene in wheat. In agreement with this review, scholars have used *Lr* gene and *Sr* gene for leaf rust and stem rust, respectively (Moore et al. 2015; Olivera et al. 2018).

Similarly, *Septoria nodorum* blotch disease has been used wrongly (Downie et al. 2021), but some of the scholars used glume blotch disease (Melville and Jemmett 1971). *Septoria nodorum* is the causal agent of glume blotch disease (Lillemo et al. 2011; Salgado and Paul 2016; Scala et al. 2020). For few years, genus *Stagnospora* was also used. *S. nodorum* was replaced by *Parastagnospora nodorum* and *Parastagnospora avenae* f. sp. *triticea* (Nedyalkova et al. 2019). As a result, Nedyalkova et al. (2019) suggested that *P. nodorum* and *P. avenae* f. sp. *triticea* are responsible for the glume blotch disease. *S. nodorum* was differentiated from *S. tritici* based on the length-to-width ratio of their conidia; the first two species (*S. nodorum* and *S. avenae* f. sp. *triticea*) are different from *S. tritici*. According to Cunfer and Ueng (1999), conidia of the genus *Septoria* may be 10 times longer than broad compared to those in the genus *Stagonospora*. Nedyalkova et al. (2019) used internal transcribed spacer (ITS) and suggested the 100% similarity of the *P. nodorum* and *P. avenae* f. sp. *triticea* species.

In another study, the species of *Z. halophila*, *Z. pseudotritici*, *Z. tritici*, *Z. ardabiliae*, and *Z. brevis* have been reported in *Zymoseptoria* genus (Stukenbrock et al. 2012). Of them, *Z. ardabiliae* and *Z. pseudotritici* have been isolated from *Elymus repens*, *Dactylis glomerata*, and *Lolium perenne* in Iran (Stukenbrock et al. 2012). As a heterothallic ascomycete, the teleomorph of *Z. tritici* is *Mycosphaerella graminicola* (Hunter et al. 1999; Linde et al. 2002; Zhan et al. 2003; Orton et al. 2011).

Under this review, my target is *Z. tritici* species. *Mycosphaerella graminicola* (Fuckel) (anamorph: *S. tritici* Roberge in Desmaz.) is the sexual form and is a species of filamentous fungus that belongs to kingdom Mycota, Phylum of Ascomycota, Class Dothideomycetes, in the family of Dothideaceae, Genus *Mycosphaerella*, Species *graminicola* (Orton et al. 2011).

### 2.2. Genetics and biology of the pathogen

#### 2.2.1. *Sexual reproduction of the* M. graminicola

This pathogen known as heterothallic fungi due to both sexual and asexual parts are present on the mycelia. During the sexual reproduction of *Mycosphaerella graminicola,* the spermatia and trichogynes were produced by spermatogonia and ascogonia, respectively, and a gametes formation occurs regularly in nature during each growing season (Crous 1998; Hunter et al. 1999). Genetic diversity of *M. graminicola* populations has been reported all over the world (Linde et al. 2002; Zhan et al. 2003).

**Figure 1.**
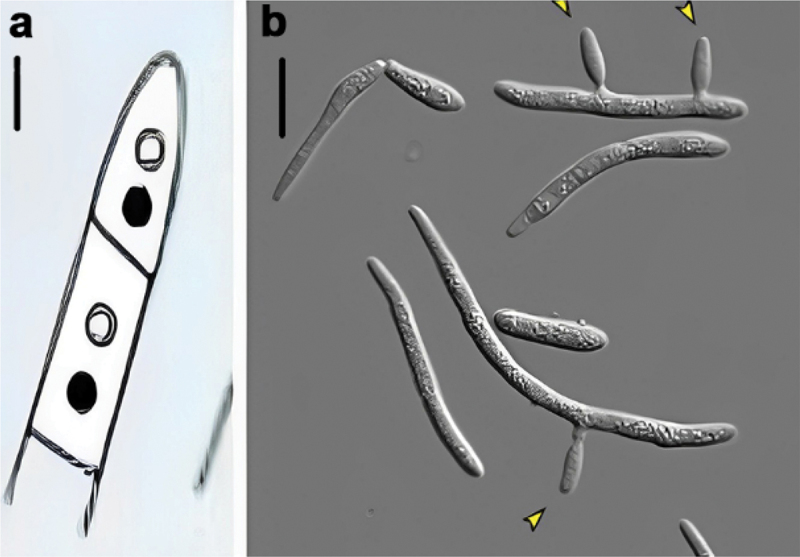
Vegetative growth forms of *Zymoseptoria tritici*. (a) Mycelium contains pair of nuclei. (b) the arrow indicates lateral budding of macropycnidiospores to form a single cell.

Plasmogamy (combination of cytoplasm): when the cytoplasm of two-parent mycelia unites, a spermatium fertilises a trichogyne. Spermatogonia gametes are produced from the male spermatia and female trichogynes (Crous 1998). The first mitosis started to divide the nuclei of spermatogonia gametes. After the first mitosis results, the septa are produced; then, the septa resulted in a dikaryotic cell having two haploid nuclei (a dikaryote) (Figure 1a).

**Figure 2.**
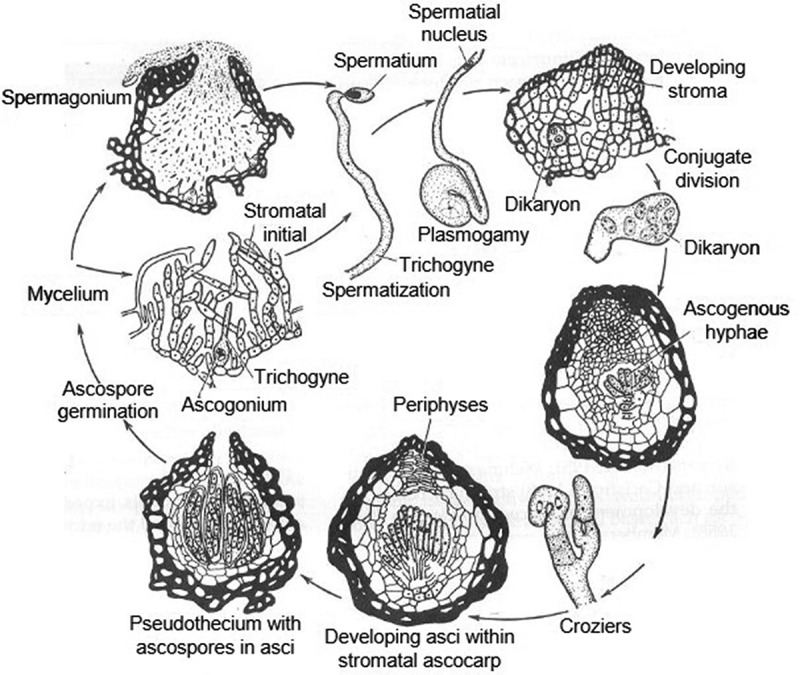
Process of sexual reproduction in *Mycosphaerella graminicola*.

Karyogamy: a combination of nuclei and zygote (nuclei fuse into a zygote); the second mitosis started to divide the nuclei of dikaryotic cell. The mitosis process resulted in ascogenous hyphae containing nuclei from each parent. From ascogenous hyphae, the ascus mother cells are produced. Then, ascus mother cells fuse into a zygote. Ascus mother cells are genetically distinct nuclei. The zygote nucleus undergoes meiosis, resulting in four haploid nuclei. Each of these nuclei will then undergo mitosis to yield four twin pairs of daughter cells (ascospores), which form within asci within fructifications called pseudothecia (Alexopoulos et al. 1996) (Figure 2).

Chromosome re-assortment and crossing over can both lead to recombination during meiosis. Re-assortment of homologous chromosomes results in the redistribution of entire chromosomes from the parents in the progeny. When non-sister chromatids of homologous chromosomes cross over, genetic material is transferred, resulting in the production of new chromosomes that include genetic material from both parents (Alexopoulos et al. 1996).

Ascocarp is the genetic name for several asci formed in the pathogen fruiting body, and it determines the pathogen sexual state. This ascocarp comes in a variety of shapes and sizes, and it can be found in various parts of the host’s tissue. One of the ascocarp forms of this disease is pseudothecia (ball shape). It is made inside lesions below the host epidermis and embedded in the tissue’s protective layer, the stroma. Pseudothecia has a dark brown globose appearance, and it is structured to produce sexual fruit. Perithecia are other form of *M. graminicola* sexual states that are flask-shaped with a pore at the tip and sub-epidermal to the plant leaf.

After the macropycnidiospore or micropycnidiospore asexual state of *M. graminicola* infected the leaves, ascospores arise (Eriksen et al. 2003). This means that *M. graminicola* sexual reproduction begins after the asexual spore germinates. Each ascus produces eight ascospores at the time of ascospore development, and hyaline (clear) comprises two cells of irregular extent (Ponomarenko et al. 2011). The sexual mating system of *M. graminicola* requires the union of two compatible partners with different mating types to create sexual spores (Sanderson et al. 1985).

The mating system is heterothallic, which means that sexual spores must be produced by two compatible partners. In reaction to the mating pheromone released by unlike mating types, two fungal strains of opposite mating types sense each other’s existence when they come together. The homothallic ones can reproduce sexually (Zhan et al. 2002). For sexual reproduction, the two compatible mating types must be there in the same geographical area at a time. Ascospore is formed when two compatible haploid nuclei of different mating types come together. When strains from several isolates that have different mating types get together, ascospores are produced (Kema and Vansilfhout 1997); hence, their occurrence is influenced by the severity of epidemics. According to Ponomarenko et al. (2011), the fungus has a bipolar, heterothallic mating system. To cause sexual reproduction, individuals of the mat1–1 and mat1–2 must come together. Sexual reproduction in *M. graminicola*, requires a physical encounter between two compatible strains (Mat1–1 and Mat1–2) (Kema et al. 1996, 2000; Zhan et al. 2002), and takes place on senescent tissues, with pseudothecia appearing 46 to 76 days after infection in field conditions (Eriksen et al. 2003; Suffert et al. 2011).

#### 2.2.2. *Asexual reproduction of the* Z. tritici

*Z*. *tritici* name was given for asexual part of mycelia of the causal agent of leaf blotch disease. It is available in three different vegetative growth forms (Sanderson et al. 1985). The first vegetative growth form of this pathogen is macropycnidiospore with three to five septa being the most common cell form. The “Yeast-like” stage of the macropycnidiospore form has been frequently mentioned (Mehrabi et al. 2006). However, yeasts are single-celled organisms, whereas macropycnidiospores are multicellular (Figure 1b) (Steinberg 2015). Individual cells in this multicellular structure range in size from 1.5 μm to 3.5 μm wide to 40 μm to 100 μm (Sanderson et al. 1985). Macropycnidiospores germinate near the apex of the plant (end). The extension of tip growth to generate thin hyphae, which are made up of highly elongated cells, is the germination process.

Micropycnidiospores are the second vegetative growth stage (Eyal et al. 1987). It has unicellular structures without septa (1 μm wide, 5 μm to 10 μm long). As a result, it fulfils the criteria of a “Yeast” growth type. Lateral budding from hyphae or macropycnidiospores produces these cells (Figure 1b) (Steinberg 2015). Another study also showed that the yeast-like growth of this pathogen. One mutant of *Z. tritici* exhibiting blastosporulation (yeast-like growth) grew fatter than the other mutant on the nutrient-rich PDA medium (Francisco et al. 2020). The fungus can be changed morphologically through switching between hyphal growth and yeast-like development as a result of conducive environment (Mehrabi et al. 2006; Motteram et al. 2011; Francisco et al. 2019).

Pycnidia are the other morphological form and asexual fruiting parts of *Z. tritici*. They are used for dissemination by rain splashes but not used for infection. They range in size from 60 μm to 200 μm, depending on the fungal strain, infection density, and wheat cultivar stomata size variations (Sanderson et al. 1985; Kema et al. 1996). The pycnidia are implanted in mesophyll and epidermal tissue on both sides of the leaf, with an aperture (ostiole) on top. The pathogen asexual condition is represented by these three types of growth.

### 2.3. Life cycle of the pathogen

After coming into touch with the host leaf, both the sexual ascospores and asexual pycnidiospores germinate and penetrate plant tissues under suitable environmental conditions (Palmer and Skinner 2002). A major source of infection for leaf blotch disease is ascospores (Cook et al. 1999). After landing on the leaf, the spores nearly completely pass through the stomata (Cohen and Eyal 1993; Duncan and Howard 2000). Moreover, the fungus initially infects the host through hyphal development through stomatal openings (Kema et al. 1996; Shetty et al. 2003). Then, it smoothly expands into the mesophyll cells with hyphae spreading out in the intercellular spaces in between the mesophyll cells. In other words, after penetrating the leaf, the fungus grows intercellularly to colonise the mesophyll cells, but it does not produce any feeding structures like haustoria (Palmer and Skinner 2002). After 3 to 11 days of infection, hyphae start to fill the substomatal space and pre-pycnidia appear in these openings (Duncan and Howard 2000; Shetty et al. 2003). The infection continues to be asymptomatic during this phase of leaf colonisation. Dead plant cells are infrequently observed, and the leaves appear healthy (Hilu and Bever 1957). This stage is called the latent phase also known as the biotrophic phase. The biotrophic phase atypically long might last between 6 and 36 days, depending on the wheat genotype-fungal isolate combination (Ponomarenko et al. 2011).

The fungus quickly transit to necrotrophic development associated with disease lesions on the leaf surface 10 days after inoculation (dai) (Shetty et al. 2003). In other words, the crop cells collapse as hyphae grow intercellularly and transition from a biotrophic to a necrotrophic state, leaving behind yellow lesions or blotches. The host cell walls have not been known to be broken, because any specialised penetrations or feeding structures have not been present for *Z. tritici* (Kema et al. 1996; Shetty et al. 2003). Throughout the whole infection cycle, it stays in the apoplastic area to collect nutrients (Rohel et al. 2001). When cells break, necrotic areas of lesions release conidia into the gelatinous hygroscopic cirrhi, and pycnidia appear at necrotic locations (Eyal et al. 1987; Ponomarenko et al. 2011). When the procedure is complete, the pycnidia mature and return to the inoculums (Ponomarenko et al. 2011) (Figure 2).

### 2.4. Ecology of the pathogen

On stubble, *Z. tritici* sustains from one season to the next. Windborne spores (ascospores) are discharged from fruiting bodies (perithecia) stayed in the stubble of previously infected plants in late fall and early winter after rain or heavy dew (Ponomarenko et al. 2011). These spores have a long range of dispersal (Chen et al. 1994). It can exist in seeds, straw, stubble, volunteers and debris, exogenous debris, adult plants, and grass species.

Once more, wheat seedlings that have grown on other wheat residues help the fungi survive. They can be discovered as pycnidia on wheat residues from one crop to the next or as mycelium in straw plants. Up to 3 years, the fungi can survive in the wheat stubble on the soil’s surface. Debris from severely diseased leaves and stems remain in fields after harvest to serve as inoculum for the following growing season (Eyal et al. 1987; Ponomarenko et al. 2011). The fungus typically overwinters in defective volunteer wheat seed, infected wheat straw from past crops, and other susceptible grasses from year to year. The fungi grow in contaminated straw and can survive for up to a year in seed form.

### 2.5. Epidemiology of the pathogen

Leaf blotch disease of wheat epidemics is linked to favourable weather (regular rainfall and moderate temperatures) (Campbell and Madden 1990), specific cultural techniques (Lovell et al. 2004), inoculum availability, and the presence of susceptible wheat cultivars.

#### 2.5.1. Conducive environment

The latent period can last from 14 to 21 days at an optimal temperature (15°C to 20°C) to 40 days at 5°C, depending on the cultivar and environmental conditions including temperature and leaf moisture (Eyal et al. 1987; Shaw 1990). *Z. tritici* infection is temperature-dependent and necessitates wet, chilly surroundings. If the right circumstances are present, an infection can happen at any stage of a plant’s development.

Because of the temperature and moisture needs, *Z. tritici* infections are most likely in the early to mid-season, when temperatures are at their coolest. Infections normally begin at the base of the plant and progress upwards until high temperatures become a limiting factor. Temperatures of 15°C to 20°C are favourable for *Z. tritici* activity. Wet and windy weather favours epidemics of leaf blotch disease due to its specific temperature and moisture needs, but dry weather generally slows or stops growth (Lucas 1998).

From the lower leaves of the wheat, rain-splattered pycnidiospores move vertically up to the higher leaves. Leaf blotch disease progression from lower infected leaves to the upper plant section is frequently disrupted by long rain intervals with high temperatures (Eyal et al. 1987). When humidity is high and there is free water on the leaf, secondary spread from conidia/ascospore is effective for epidemics. The quantity of ascospores emitted is influenced by the length and intensity of rain, temperature, and wind (Lovell et al. 2004).

#### 2.5.2. Susceptible materials of wheat

The usual vertical progression of leaf blotch disease from lower to upper leaves is impacted by the “ladder effect.” The first three to four developing leaves on both short and tall cultivars are equally spaced; however, on tall varieties, the distance between each leaf increases towards the flag leaf (Shaw 1990; Shaw and Royle 1993; Lovell et al. 2004).

The proximity of the upper leaves to the lower leaves in dwarf cultivars (70 cm to 90 cm) enables contact between freshly developing leaves and splashing pycnidiospores. The studies on leaf blotch disease indicated that dwarf cultivars with higher plant parts are more susceptible to the disease than taller wheat because they are closer to inoculum sources (Eyal 1971). Thus, it is made easier for the disease to spread from infected lower leaves. Pycnidia consequently appear earlier on the higher plant portions of dwarf cultivars than they do on the leaves of taller cultivars. As a result, both resistance and morphology-related genetic variables have an impact on leaf blotch disease spread and severity (Lovell et al. 2004).

As indicated in several crop disease studies, crop architecture is being used to increase disease resistance. Numerous studies have discovered that some architectural features enable pathogens to escape by preventing the pathogen from making direct contact with the host, creating an unfavourable environment for the disease, or inhibiting the pathogen’s ability to start an infection (Ando et al. 2007). Leaf positioning should be considered when new wheat cultivars are issued in wheat-growing locations where leaf blotch disease is a possible threat (Shaw and Royle 1993; Pietravalle et al. 2003; Lovell et al. 2004). According to the same theory, disease severity is significantly influenced by plant stance (Saindon et al. 1995), growing conditions (Ando and Grumet 2006), and plant height (Hilton et al. 1999). Numerous factors have been shown to affect disease severity (Ando et al. 2007), including the spatial distribution of leaf area in the canopy (Schwartz et al. 1978), the number of leaves (Jurke and Fernando 2008), and leaf shape (Gan et al. 2007).

With a slower stem extension, the leaf blotch disease has more time to spread from older to younger leaves (Shaw and Royle 1993; Lovell et al. 2004). Furthermore, in diseased plants, too long a maturation time results in another *Z. tritici* cycle of proliferation (Shaw and Royle 1993). Leaf blotch disease resistance was also found to be inversely correlated with plant height using quantitative trait locus (QTL) analysis. As a result, the plant height-controlling Rht-D1 gene has been found to have a considerable effect on leaf blotch disease resistance (Miedaner et al. 2012). Negative correlations of leaf blotch disease resistance with plant height and heading date were found in groups where Rht-D1 was not segregating (Risser et al. 2011; Miedaner et al. 2012; Andersson et al. 2022). However, it was discovered that tall and late wheat genotypes are less vulnerable to *Z. tritici* infection than short and early wheat genotypes. The co-localisation of QTL for leaf blotch disease resistance and dwarfing gene loci (Baltazar et al. 1990; Risser et al. 2011), in addition to additional plant height QTL (Eriksen et al. 2003), supported this morphological resistance at the gene level.

At the seedling growth stage, many bread and durum wheat-susceptible materials have been reported (Medini and Hamza 2008; Makhdoomi et al. 2015; Mekonnen et al. 2020; Ababa et al. 2022; Burcu et al. 2022). Shafir has *Lb6* resistant gene, and Estanzuela Federal has *Lb7* resistant gene; however, they did not show resistance reaction to any of the tested isolates (Makhdoomi et al. 2015). Mekonnen et al. (2020) also stated that commercial cultivars like Laketch, Et-13A2, Gassay, Dembal, Tay, Ogolcho, Kakaba, King Bird, and Millinum were susceptible to leaf blotch disease, in addition to the differential lines Salamouni, Veranopolis, Israel-493, Tadinia, Shafir, Estanzuela Federal, Kavkaz-K4500, and Km7. Additionally, K6295-4A, LEMU, Yerer, Laketch, and ET-A132 are only a few of the commercial wheat cultivars that were identified as being susceptible (Ababa et al. 2022). Both durum and bread wheat were examined at the adult growth stage for susceptibility. According to Kidane et al. (2017), Tossa line was more susceptible both at heading and maturity on the severity of leaf blotch disease. The most vulnerable genotypes at heading, mid-maturity, and maturity phases were EBW104 (39.8%), EBW076 (48.5%), and Paven-76 (65.3%) (Mekonnen et al. 2020).

#### 2.5.3. *Virulent isolates or genetic variability of* Z. tritici

The sexual reproduction of *Z. tritici* regulates both the variety of regional populations and the quantity of primary inoculum (wind-dispersed ascospores) available to trigger subsequent epidemics (Suffert et al. 2011). Different virulent *Z. tritici* isolates from multiple countries have been reported (Grieger et al. 2005). The Canadian (Can1, Can2, and Can3) isolates were virulent on the same differential lines, except on the differential line has *Lb6* gene (Medini and Hamza 2008). In another country, RM155 isolate was virulent on 14 out of 18 differential lines (Makhdoomi et al. 2015). K-5 isolate from the Konya province displayed virulent reaction on 10 of the 12 wheat differential set (Burcu et al. 2022). Mekonnen et al. (2020) also reported that the I3 isolate in Ethiopia was virulent on all differential lines, including Salamouni, Veranopolis, Israel-493, Tadinia, Shafir, Estanzuela Federal, Kavkaz-K4500, and Km7. Later, EtA-19 which was virulent on the Salamouni, Veranopolis, Israel-493, Tadinia, Estanzuela Federal, Kavkaz-K4500, and Km7 differential lines was reported (unpublished data).

The diversity of *Z. tritici* isolates has been reported so far. Hosseinnezhad et al. (2014) discovered that Iranian *Z. tritici* isolates showed significantly different virulence patterns, with virulence spectra ranging from 40% to 90% of the tested genotypes. Eight Iranian *Z. tritici* isolates were tested on 45 durum wheat landraces, and the isolate pathogenicity spectrum ranged from 42% to 82% (Ghaneie et al. 2012). Ghaneie et al. (2012) found that eight Iranian *Z. tritici* isolates tested on 45 durum wheat landraces had a wide virulence spectrum. The isolates were virulent from 42% to 82% of the wheat genotypes evaluated. In another country, Burcu et al. (2022) identified 28 pathotypes from 60 isolates in four provinces of the central Anatolia region of Turkey. Burcu et al. (2022) detected a broad spectrum of virulence for the *Z. tritici* isolates using the differential wheat lines; from these pathotypes, they observed 47% pathogenic diversity of Turkey isolates. Medini and Hamza (2008) identified pathotypes of *Z. tritici*. Therefore, they suggested 19% pathogenic diversity of Tunisia, Algeria, and Canada isolates from eight pathotypes detected. Mekonnen et al. (2020) found *Z. tritici* to have a broad-spectrum virulence, successfully infecting 71% to 93% of the differential lines. Currently, in Ethiopia, out of 43 isolates, 25 pathotypes, were identified based on the pycnidia percent parameter; this are implying 58.1% pathogenic diversity of isolates (unpublished data).

Moreover, the diversity of the virulent gene in this pathogen has been reported. The sequencing of *AvrLb6* from populations of *Z. tritici* had been done in two earlier investigations. To examine the diversities of *AvrLb6*, some of the scholars collected a global population of *Z. tritici* between 1990 and 2001 (Zhan et al. 2005; Brunner and McDonald 2018); moreover, other populations collected from France in 2009–2010 (Zhong et al. 2017). A wide variety of *AvrLb6* haplotypes were discovered, along with proof of positive selection brought on by point mutations and recombination. *AvrLb6* was discovered to be positioned in a very dynamic area of *Z. tritici* chromosome 5, where there is significant transposon activity that contributes to *AvrLb6* polymorphism (Sánchez-Vallet et al. 2018).

Although the *AvrLb6* haplotype distribution in these older collections has been extensively defined, it is uncertain how diverse the *AvrLb6* haplotypes are in more recent *Z. tritici* populations. The changes at two amino acid residues (positions 41 and 43) in the *AvrLb6* protein have been suggested as being critical for the pathogenicity of wheat cultivars carrying *Lb6*, the precise polymorphisms that drive the change from avirulence to virulence phenotype in the *AvrLb6* protein have not yet been identified (Kema et al. 2018). Stephens et al. (2021) analysed the diversity of the avirulence factor *AvrLb6* in the recent global *Z*. *tritici* populations. They sequenced the *AvrLb6* gene from recent field populations of *Z*. *tritici* isolates collected between 2013 and 2017. Therefore, they suggested that from the previous studies, the large shifts in *AvrLb6* haplotype prevalence have taken place in multiple global regions over the relatively short period between samplings.

### 2.6. Wheat leaf blotch disease management practices

#### 2.6.1. Cultivars mixture

Improved epidemics of pests and transferable diseases in wildlife have been linked to decreased biodiversity caused by human activities (King and Lively 2012; Ostfeld and Keesing 2012; Civitello et al. 2015). The deliberate introduction of genetic variation into populations of crop plants has been proposed as a possible counter measure for lowering outbreaks of plant diseases and enhancing crop function (Finckh and Wolfe 2006). One strategy for genetically varying agricultural plants is to simultaneously cultivate two or more genotypes of the same crop in the same location. A physical cultivar combination can be created by combining seeds from various cultivars before sowing.

The theory behind cultivar mixtures is that genetic, physiological, structural, and phonological diversity among the components of the mixture specifically, the various cultivars that make up the mixture may promote advantageous interactions not only between genotypes but also between genotypes and environments (Newton et al. 2009; Borg et al. 2018; Kristoffersen et al. 2020). Consequently, cultivar combinations have been shown to increase yield and stability relative to pure stands and increase crop resistance to biotic and abiotic stresses, particularly in low pesticide cropping systems (Smithson and Lenne 1996; Borg et al. 2018). The greater significance of this idea is that cultivar combinations can stop disease epidemics from spreading when the components of the mixture have various degrees of resistance to the targeted disease (Wolfe 1985; Finckh and Wolfe 2006; Gigot et al. 2013). If the components in the mixture are properly chosen, mixtures can also improve the quality of the final product (Finckh et al. 2000; Cowger and Mundt 2002).

Therefore, different scholars identified genetic homogeneity as a critical component in the formation of disease outbreaks in crops (Johnson 1961; Person 1966; Browning and Frey 1969). To combat this, multiline combinations have been proposed as a way to deliberately introduce variability into host populations (Jensen 1952; Borlaug 1959; Browning and Frey 1969; Wolfe and Finckh 1997).

Combining resistant and susceptible isogenic lines, cultivars, or even species may lessen the severity of epidemics by slowing the spread of secondary diseases via spores released from susceptible plants that have been affected. Combinations are particularly effective in combating wind-dispersed diseases with shallow spore dispersal gradients, according to simulation modelling. However, diseases like barley rhynchosporium, which have steep spore dispersion gradients and less obvious gene-for-gene interactions due to secondary transmission by splash-dispersed spores, have demonstrated that combinations can enable effective control (McDonald et al. 1988).

Cultivar mixes are effective against splash-dispersed pathogens such as *Z. tritici*, *R. secalis*, and *S. nodorum* (Jeger et al. 1981; McDonald et al. 1988; Newton et al. 1997). It is believed that their mode of action involves the dilution of vulnerable cultivars, the barrier effect of resistant cultivars, and induced resistance (Chin and Wolfe 1984). It is supposed that induced resistance can help control epidemics brought on by biotrophic, airborne diseases with highly specialised host–pathogen interactions (Calonnec et al. 1996). *R. secalis*, on the other hand, exhibits a genetically diverse population structure (McDermott et al. 1989). In rusts and powdery mildews, the host-pathogen connection is substantially less specific (Newton and Thomas 1993). Furthermore, in commercial cultivars, there are fewer sources of particular resistance to *R. secalis*. Cultivar combinations are therefore probably less effective against *R. secalis* than against biotrophic diseases. However, non-specific resistance can be used in cultivar mixtures to reduce infection (Jeger et al. 1981; McDonald et al. 1988; Mundt et al. 1994).

Some three-component mixtures with two susceptible and one resistant lines, as well as some two-component mixes with one resistant and one susceptible line, did not exhibit any greater scald disease than the resistant component of the mixture grown alone (McDonald et al. 1988). When compared to pure stands, cultivar combinations reduced leaf blotch disease by 10.6% (Kristoffersen et al. 2020). The susceptible pure stands received only 25% of a resistant component, which led to a surprising 48% reduction in disease levels (Ben et al. 2020). Different leaf blotch disease resistance scores, similar earliness, and plant height, as indicated by several trials, were taken into consideration while selecting the treatment arrangements. Their research suggests that the testing should be conducted using pure stands, two-way mixtures, and three-way mixtures (Table 1). The ratio of resistant cultivars to susceptible combinations was always 25%, 50%, or 75% (Ben et al. 2020). The cultivar proportion suggested by different scholars was reviewed (Ababa et al. 2023).

**Table 1.** Cultivar proportions have been employed in pure stands, two-way mixtures, and three-way mixtures planted in three replicates.

Pure stand					Two-way interaction						Three-way interaction		
T	5	1	6	14	2	3	4	8	9	10	11	12	13
**Bread wheat cultivars mixture**													
T1	100				25	50	75	25	50	75	25	50	75
T2		100			75	50	25				37.5	25	12.5
T3			100					75	50	75	37.5	25	12.5
T4				100									
**Durum wheat cultivars mixture**													
T1	100				25	50	75	25	50	75	25	50	75
T2		100			75	50	25				37.5	25	12.5
T3			100					75	50	75	37.5	25	12.5
T4				100									

T is treatment, T1 (%) is treatment one (Susceptible), T2 (%) is treatment two (Resistant), T3 (%) is treatment three (Resistant), and T4 (%) is treatment four (Resistant).

#### 2.6.2. Resistant materials of wheat

During leaf blotch disease infection, wheat additionally secretes small proteins into the apoplast to aid in the detection of *Z. tritici* proteins and/or to elicit defence responses. Nuclear-binding leucine-rich repeat (NB-LRR) proteins externally bind to pathogen recognition receptors (PRRs) on the wheat cell surface to recognise *Z. tritici* pathogen-associated molecular pattern (PAMP) chitin after infection and start the effector-triggered immunity (ETI). Wheat has evolved a multi-layered immune system to recognise and resist *Z. tritici* (Jones and Dangl 2006). ETI or pathogen-associated effector-triggered immunity is the initial layer of plant immunity. A hypersensitive reaction to effector recognition that results in ETI is sometimes accompanied by salicylic acid (SA) signalling and systemic acquired resistance (SAR) (Kombrink and Schmelzer 2001). A growing portion of the wheat called apoplast acts as a barrier between the wheat and *Z. tritici*. For ETI, it is the area outside the plasma membrane (Block et al. 2014; Jashni et al. 2015; Wang and Wang 2018; Schellenberger et al. 2019).

Wheat can activate genes involved in pathogenesis-related (PR), including those involved in the generation of reactive oxygen species (ROS) and the activation of transcription factors. Once more, it secretes a variety of PR proteins into the apoplast that have been shown to hydrolyse glucans, chitin, and polypeptides (Ilyas et al. 2015; Ali et al. 2018), inhibit pathogen-secreted enzymes (Jashni et al. 2015; Wang et al. 2017), and phytochemically inhibit pathogen growth (Wirthmueller et al. 2013). Small secreted proteins (SSPs) are effectors that *Z. tritici* can exploit to stop or lessen ETI-induced defence responses (Palma-Guerrero et al. 2016). Comparative genomes and transcriptome studies have led to the identification of numerous possible *Z. tritici* effector genes (Gohari 2015; Rudd et al. 2015; Palma-Guerrero et al. 2016; Kettles et al. 2017; Plissonneau et al. 2018; Zhou et al. 2020).

For all of these defence mechanisms of wheat to leaf blotch disease, several models have been utilised; invasion or spatial invasion model is one of them, the condensed form reaction against apoplastic leaf pathogens (Stotz et al. 2014; Cook et al. 2015; Kanyuka and Rudd 2019). For this invasion model, different *Lb* genes have been cloned, and the resistance gene was introduced into wheat. *Lb16q* is the second *Lb* gene cloned, that is responsible for broad-spectrum resistance, and it encodes a cysteine-rich receptor-like kinase (Saintenac et al. 2021). *Lb16q* and *Lb17* are two *Lb* genes found in Synthetic M3 that are responsible for broad-spectrum resistance. According to earlier studies, *Lb17* is effective only at adult plant resistance (Tabib Ghaffary et al. 2012), indicating that *Lb16q*, which is known to confer broad resistance against *Z. tritici*, is principally responsible for Synthetic M3 resistance. It should be highlighted that the resistance offered by *Lb16q* in the field is probably less complete. The field effectiveness of *Lb16q* will likely decline over the following years (as was the case with *Lb6* and *Lb15* previously) due to the selection of *Z. tritici* isolates with virulence against the line carrying *Lb16q* resistance gene (Dalvand et al. 2018; Kildea et al. 2020). When important resistance genes break, agricultural systems are vulnerable because wheat lacks broad-spectrum leaf blotch disease resistance. Both *Lb6* and *Lb15* have been extensively utilised in Northern Europe and were initially very effective; however, because of the selection pressures brought up by their extensive usage, *Z. tritici* has now extensively broken both of them (Chartrain et al. 2004; Arraiano et al. 2009; Stephens et al. 2021).

It has been demonstrated that Kavkaz-K4500, one of the most reliable sources of field resistance utilised in breeding, possesses at least five qualitative resistance genes, including *Lb6*, *Lb7, Lb10*, and *Lb12* (Chartrain et al. 2005). Although many international *Z. tritici* isolates are virulent on it in laboratory tests (Chartrain et al. 2004, 2005), this combination of *Lb* genes appears to be sufficient to make Kavkaz-K4500 resistant to leaf blotch disease under field conditions. This may indicate high genetic diversity differences between UK and international *Z. tritici* populations or could be related to the different levels of inoculum used in laboratory vs field trials.

Additionally, some lines including TE9111 (containing *Lb6*, *Lb7*, and *Lb11*) and Lorikeet (containing *Lb19*) were resistant to pycnidia production from every *Z. tritici* strain (Tidd et al. 2023). Breeders should be most interested in these lines. Previous studies indicated that *Lb6* and *Lb7* genes did not show resistance to UK *Z. tritici* populations; therefore, *Lb5*, *Lb11*, and either *Lb10* or *Lb12* are responsible (Czembor et al. 2011; Makhdoomi et al. 2015; Stephens et al. 2021). Currently, *Lb5* and *Lb11* appear to be the optimal resistances to protect the durability of *Lb19* in future wide use (Tidd et al. 2023).

Black and Gallegly (1957) described field resistance (also known as horizontal resistance). Eyal et al. (1985) proposed that KK variety resistance could be governed by up to seven genes. Other lines include TE 9111, Veranopolis, and Olaf have four, whereas Chaucer and Catbird have two resistant genes (Chartrain et al. 2004). KK variety has been used as a source of resistance to leaf blotch disease for many years and is one of the most resistant in the field (Eyal et al. 1985; Kema and Vansilfhout 1997; Arraiano et al. 2001). Again, Chartrain et al. (2004) suggested the most resistant line was Senat, followed by Gene, Milan, Israel 493, and Chaucer.

Germplasm from China (Synthetic 6×), Latin America (SO852), and Europe (Arina, and Shafir) has been identified as potential resistance sources (Arraiano et al. 2001, 2007; Adhikari et al. 2004; Chartrain et al. 2004). However, several sources are not suitable for commercial breeding (Arraiano et al. 2001, 2007; Adhikari et al. 2004; Chartrain et al. 2004). Makhdoomi et al. (2015) proposed five wheat genotypes for breeding efforts, all of which were resistant to the six isolates tested in Iran. According to (Mekonnen et al. 2020), the Hidase cultivar displayed three to five resistance levels in Ethiopia. Danda’a, HONQOLO, Digalu, Dashen, EJERSA, Alemtena, Mosobo, Hitosa, Robe, and Lelisso materials were proposed as broad-spectrum materials (Ababa et al. 2022).

The adult plant resistance of wheat crop against leaf blotch disease has been discovered by many investigators. Genome-wide association study (GWAS) has been successfully used to mine multiple putative QTLs/genes related to agronomically important features in a variety of plants, including disease resistance (Bartoli and Roux 2017; Kidane et al. 2017; Juliana et al. 2018; Odilbekov et al. 2019; Alemu et al. 2021; Mekonnen et al. 2021). For leaf blotch disease, several valuable QTLs/genes have been discovered by GWAS and linkage mapping (Schilly et al. 2011; Tabib Ghaffary et al. 2012; Miedaner et al. 2013; Dreisigacker et al. 2015; Mirdita et al. 2015; Muqaddasi et al. 2019; Odilbekov et al. 2019).

Again, adult plant resistance was detected at wheat heading, mid-maturity, and maturity growth stages (Dreisigacker et al. 2015; Kidane et al. 2017; Muqaddasi et al. 2019; Alemu et al. 2021; Mekonnen et al. 2021). They proposed that the disease was most severe at the maturity growth stage, and the response of each genotype was considerably different at each stage. Selam showed lower leaf blotch disease severity at the heading and maturity growth stages. Mangudo expressed lower leaf blotch disease severity at maturity and heading growth stages. Therefore, both cultivars have more resistance than other cultivars (Kidane et al. 2017). EBW174 showed resistance at all growth stages having a leaf blotch disease severity of 5.3% (Mekonnen et al. 2020). Some genotypes performed better for leaf blotch disease resistance than others. Borstvete was more resistant than Gotland and Ankar. Again, Hereford was more resistant than Sweden and Aring from Denmark (Alemu et al. 2021). When compared to the line IAS20 × 5/H567.71, RPB709.71/COC parent displayed greater resistance. In the CIMMYT wheat lines IAS20 × 5/H567.71 and RPB709.71/COC, there are a total of five consistent QTL for leaf blotch disease resistance across all environmental conditions (Dreisigacker et al. 2015).

A genome-wide association (GWA) scan utilising SDS data obtained at the heading showed likely QTLs on chromosomes 1D, 2A, 3A, 3D, 5A, 7A, and 7D. Therefore, 33 potential quantitative trait loci (QTLs) were discovered in bread wheat (Mekonnen et al. 2021). Furthermore, effective putative QTLs on chromosomes 1B, 3D, and 7B were revealed in the association analysis for SDS at the mid-maturity growth stage. Similarly, at the maturity stage, potential QTLs on chromosomes 1D, 4A, and 6A were discovered. On durum wheat, the GWA scan identified five significant potential QTL for leaf blotch disease resistance (Kidane et al. 2017).

After, Flor (1942) reported the gene for gene hypothesis in flux and rust pathosystem, different scholars reported this type of resistance in different crop pathosystems. The specificity in *Z. tritici* and wheat pathosystems had been debated for more than 20 years. Then, in 1973, the specificity of this pathogen was confirmed (Eyal et al. 1973). Of 89 genomic regions, 27 had been detected at the seedling growth stage. Moreover, 22 *Lb* genes have been observed for specific resistance (Brading et al. 2002; Brown et al. 2015), including 12 isolate-specific genes and 10 non-isolate-specific genes from wheat (Ghaffary et al. 2018; Yang et al. 2018).

*Lb6* is the one of *Lb* gene cloned that is responsible for race-specific resistance and it encodes a wall-associated-like receptor kinase (Zhong et al. 2017; Kema et al. 2018; Saintenac et al. 2018). *Lb1* is the first; it is available to growers in the cultivars Oasis and Sullivan in 1975. The efficacy of this gene had been used for 3 years (Cowger et al. 2000; Adhikari et al. 2004; Singh et al. 2016). Saw is a cross between Tadorna, Cleo, and Inia 66, then released in 1984 with *Lb4* gene (Somasco et al. 1996). *Lb4* also demonstrated a respectable level of durability, lasting approximately 15 years. The Cougar variety was released in 1992, but it had served for 3 years (Cowger et al. 2000), due to Cougar-virulent strains of *Z. tritici* in the UK (Kildea et al. 2021).

In general, *Lb4* and *Lb6* genes have lost their resistance due to *Lb6q avr* gene (Brading et al. 2002; Adhikari et al. 2004; Stephens et al. 2021), whereas *Lb*2/11/WW and *Lb18* genes have lost their resistance due to other new virulent isolates (Tabib Ghaffary et al. 2011; Liu et al. 2013; Dreisigacker et al. 2015).

The majority of commercial wheat cultivars are susceptible to the disease despite the discovery of 22 *Lb* resistant genes. Other scholars discovered 17 significant resistance genes (*Lb* genes) (Goodwin 2007; Ghaffary 2011; Orton et al. 2011). To date, 60 major leaf blotch disease resistance genes have been mapped and found, ranging from *Lb* 1 to *Lb* 22 for race specific resistance (Brading et al. 2002; Brown et al. 2015). Today commercially available varieties are largely partially resistant to leaf blotch disease (i.e. they are moderately susceptible).

#### 2.6.3. Cultural practices

In the concept of crop rotation, prevention of planting wheat in farmlands with high levels of stubble-borne inoculum is very crucial (Eyal et al. 1987; Ponomarenko et al. 2011). Two to three years of crop rotation, tilling, and the eradication of volunteer are very important to reduce the leaf blotch disease. At different times, a disease outbreak can be achieved with a one-year rotation. Furthermore, Pedersen (1992) discovered that a 1-year break between wheat crops was effective in reducing leaf blotch disease severity under unfavourable environmental conditions, whereas a 2-year crop rotation between wheat crops was necessary under ideal environmental conditions. However, during extremely dry seasons, the fungus can persist on stubble for up to 18 months (Bankina et al. 2014). Krupinsky (1999) recommended crop rotation as a technique to hasten the breakdown of infected crop residue, while non-host crops were being grown. The author suggested that crop rotation will lower the pathogen inoculum level but not eradicate the disease. However, some findings claim that crop rotation does not affect leaf blotch disease. Tan spot severity was significantly higher in repeated wheat sowings; however, crop rotation did not affect leaf blotch disease development (Bankina et al. 2014).

As the frequency of ploughing increased, the severity of leaf blotch disease was reduced (Bailey et al. 2001; Gilbert and Woods 2001; Bankina et al. 2014; Fernandez et al. 2016; Ababa et al. 2021). Numerous studies on the effect of soil tillage on leaf blotch disease have been conducted. Despite the contradicting findings, conventional tillage-ploughed plots had a higher severity of leaf blotch disease than plots with alternative tillage techniques (Gilbert and Woods 2001; Krupinsky et al. 2007; Bürger et al. 2012).

Surprisingly, Huber et al. (1987) in Indiana discovered that the intensity of tan spots on wheat cultivars was reduced as the N rate increased. However, it has been noted that using a lot of N fertiliser can make the severity of leaf and glume blotch infections on winter wheat worse (Broscious et al. 1985; Ditsch and Grove 1991; Howard et al. 1994). Depending on the geography and the local environment, increasing N fertiliser rates either appear to have a positive, negative, or no effect on the severity of the leaf blotch disease (Krupinsky 1999). As N fertiliser increased, the plant height was also increased, therefore resulted in the reduction of leaf blotch disease severity. In reverse, as nitrogen increased, the tiller number was also increased and then resulted in the increments of the leaf blotch disease severity (Krupinsky 1999). Leaf blotch disease levels can also be reduced by wheat traits such as taller plant height and late heading date or flowering time that contributes to disease escape (Simó et al. 2004; Arraiano et al. 2009; Brown et al. 2015).

Studies on crop diseases indicate that cropping practices, such as nitrogen fertilisation, planting density, and sowing date, have a variety of effects on disease development, including changes to the canopy’s architectural structure. However, these effects are intermittent, and it may not be clear how to interpret them. According to some authors, increasing planting density caused the foliar disease to occur more frequently (Ando and Grumet 2006; Gan et al. 2007; Jurke and Fernando 2008).

The development of a favourable microclimate, such as elevated relative humidity, or modifications in canopy architecture was thought to be the causes of the density effect (Tompkins et al. 1993). According to Pielaat et al. (2002), denser canopies increase leaf-to-leaf contact and facilitate the spread of disease through the canopy. The substantial increase in wheat tiller numbers with increased plant density may facilitate the deposition of *Z. tritici* spores (Broscious et al. 1985). However, there are some contradictions in the literature when it comes to the density impact. Pfleeger and Mundt (1998) discovered that plant density has only a little impact on disease development. Less dense plant stands had a sparser canopy as a result of lower plant densities, which increased the chance of rainfall splashing on lower leaves and accelerated the spread of disease (Eyal 1981).

According to various scientists, the planting date has a significant impact on disease (Shaner et al. 1975; Thomas et al. 1989; Shaw and Royle 1993; Hailemariam et al. 2020), and the crops planted early in the season have a higher risk of infection. After a leaf blotch disease outbreak, postponing of wheat early planting is very important since a lot of ascospores are discharged early in the season. Early-planted crops are more susceptible to infection. This could be early-sown plants develop more leaves, which means more inoculums are present (Shaw and Royle 1993). Furthermore, with early-sown crops, the infection has more time to migrate from older to younger leaves due to the slower stem expansion (Shaw and Royle 1993; Lovell et al. 2004). Because early-sown plants have a lot of leaves, early-planted fields might have more disease activity (Shaw and Royle 1993), which leads to a higher occurrence of inocula (Shaner et al. 1975; Shaw and Royle 1993).

Some plants are only susceptible to a pathogen at a specific growth stage (young leaves, stems, or fruits; blossoming or fruiting; maturity and early senescence); as a result, if the pathogen is absent or inactive at this specific stage, such plants avoid infection, though the latent infection may occur (Agrios 2005).

#### 2.6.4. Biological control

Global populations of *Z. tritici* have developed resistance to the most frequently employed fungicides, including azole and quinone outside inhibitors (QoI) (Fraaije et al. 2005). To combat the rise in fungicide resistance, alternative control measures including biological control are becoming increasingly important. Despite these obstacles, only a small portion of the pesticide industry, which is still dominated by synthetic chemicals, uses safe and environmentally acceptable plant protection methods (Lynch et al. 2016).

Biological control agents are being developed as an alternate control approach. Treatments for biological control include living microorganisms or abiotic substances that can (i) protect plants by creating antibiotics or other compounds that hinder the growth of pathogens; (ii) compete with pathogens for nutrients and space; (iii) cause plant resistance. Few studies have been reported on the use of *Paecilomyces lilacinus*, *Nigrospora sphaerica*, *Cryptococcus* sp., *Bacillus* sp. or their metabolites as leaf blotch disease control agents (Perello et al. 2002; Kildea et al. 2008).

According to Lynch et al. (2016), biological organisms such as lactic bacterial strains have a strong inhibitory action against leaf blotch disease. They also suggested that LAB can stop the leaf blotch disease from growing. *Lactobacillus brevis* JJ2P, *Lactobacillus arizonensis* R13, and *Lactobacillus reuteri* R2 suppressed as seen by massive mycelium on modified MRS (De Man, Rogosa, and Sharpe) agar. Lactic acid bacteria (LAB) were used as natural biocontrol agents in a variety of foods and feeds (Stiles 1996; Carr et al. 2002; Schnürer and Magnusson 2005; Broberg et al. 2007). Antifungal activity of LAB has been demonstrated against an extensive range of fungi (Corsetti et al. 1998; Stiles et al. 2002). Again, various data suggested that *Trichoderma* spss was found to have the ability to control the growth and severity of leaf blotch disease. *Trichoderma harzianum* and *Gliocladium roseum* were used as biological controls in the greenhouse and in vitro. Therefore, the severity of leaf blotch disease was significantly reduced by these microbes (Perelló et al. 1997) but, *T. harzianum* reduced *Z. tritici* growth more effectively than *G. roseum*. Both biocontrols were capable of completing and covering the colony expansion of leaf blotch disease. Some studies showed that lipopeptides from *B. subtilis* controlled *Z. tritici*. Therefore, as *Z. tritici* treated by mycosubtilin alone or in mixture with surfactin or with both surfactin and fengycin up to 82% reduction was resulted (Mejri et al. 2018). *Bacillus subtilis* ATCC 10,783, *B. cereus* ATCC 11,778, *B. licheniformis* NRRLB-510, *B. pumilus* ATCC 7061, *Brevibacillus laterosporus* BLA170, and *Paenibacillus polymyxa* NA are some of the microbes recommended for *Z. tritici* management (Alippi et al. 2000; Dutilloy et al. 2022).

#### 2.6.5. Fungicides

Fungicides are the principal way of disease control during the lack of high-resistance wheat cultivars and high disease pressure. The use of foliar fungicide sprays and seed treatment can both provide chemical control. To manage leaf blotch disease, three main fungicide groups have been suggested: Succinate dehydrogenase inhibitors (SDHI), 14a-demethylase inhibitors (DMIs), and Quinone outside inhibitors (QoI).

Since many years ago, compounds from those three families have been used effectively (Mäe et al. 2020). 14a-demethylase inhibitors (DMIs), like epiconazole and prothioconazole, and Sterol 14-demethylation inhibitors (DMI), also known as triazoles, are a type of sterol biosynthesis inhibitor (SBI). The other family is Succinate dehydrogenase inhibitors (SDHI) like Fluxapyroxad, isopyrazam, bixafen, and biscalid (Fraaije et al. 2012).

Due to Benzimidazole fungicides (Garnault et al. 2019) and quinone outside inhibitors (QoIs) (Fraaije et al. 2005) resistance developed, systemic demethylation inhibitors (DMIs; azole fungicides) and the protective multi-site inhibitor chlorothalonil have been recommended. Again, azole fungicide resistance developed in *Z. tritici* populations, field performance of several products has been negatively impacted (Clark 2006). Therefore, further changes in susceptibility to various azoles due to the ongoing evolution of novel CYP51 (sterol 14ademethylase) variations (Brunner et al. 2008; Cools and Fraaije 2008; Cools et al. 2011; Leroux and Walker 2011), and guarantee long-term sustainable disease management, new modalities of intervention are urgently required. Moreover, sensitivity in *Z. tritici* populations has been declining over time (Clark 2006). Recently, carboxamide fungicides such as boscalid (2005), isopyrazam (2010), and bixafen (2011) are entered the global cereal market. These fungicides inhibited succinate dehydrogenase (Sdh) of the mitochondrial respiratory chain (Mäe et al. 2020).

Various scientists have proposed that the criteria should be considered when utilising fungicides. The tan spot spread quickly soon after flowering. Therefore, early sprayings are ineffective under these circumstances, and these factors are essential in determining the best time to employ fungicides (Bankina and Priekule 2011). Other scholars have discovered similar patterns: After growth stage 59, leaf necrotic spots appeared quickly, and a single fungicide handling was effective (Wyczling et al. 2010). Wegulo et al. (2009) established that leaf blotch disease (induced by many diseases) developed rapidly just after flowering, with disease severity growing exponentially until milk maturity. At the flowering stage, the severity of the disease had the largest association with yield reductions (Wegulo et al. 2009).

The time of fungicide applications will be crucial for achieving effective disease control in high-risk areas. In early-sown susceptible varieties, a fungicide application at development stages 31 to 32 may be necessary to control the disease and protect new leaves. Once the flag leaf has fully developed at GS39, another application of fungicide may be necessary to safeguard the upper canopy. To find the best fungicide treatment options, numerous studies have been undertaken all around the world. Because lower fungicide doses are not allowed, research must focus on establishing the best time to spray and disease damage thresholds. Compared to one application at GS 31 or GS 39, two applications of trifloxystrobin propiconazole (at GS 31 and again at GS 39) reduced disease severity and AUDPC while increasing yield. This was a done deal because the two applications extended the period of disease control compared to the single application (Wegulo et al. 2009).

Bankina et al. (2014) found that the fungicide application yielded 9.8% to 13.5% more than control treatment. Wegulo et al. (2012) established that fungicides containing strobilurins considerably increased yield, with a mean yield difference between treated and untreated regions ranging from 12.6% to 29.4%. Different fungicide spraying techniques are utilised, but for rigorous control of winter wheat, two (and occasionally three) applications are often made (Bankina et al. 2014).

Because *Septoria* spp. has evolved resistance to a variety of fungicides, it is recommended to employ SDHI fungicides in combination with a maximum of two sprays per season as a preventive measure. Fungicide-resistant isolates are less common when fungicidal compounds with diverse modes of action, such as azoles and SDHI (Fraaije et al. 2012).

There are a few approaches that have been proposed to minimise the selection rate for new mutations. The first method is to use various triazoles since *Z. tritici* fungus mutations do not affect all triazole fungicides equally. The same triazole fungicide should not be applied if many treatments are necessary for single season. The second choice is to utilise recommended fungicides such as triazoles, flutriafol, propiconazole and cyproconazole or tebuconazole and flutriafol.

Utilising fungicides with different modes of action is the third approach; however, there is a small selection of fungicides with various modes of action. Utilising solutions that combine triazole and strobilurin fungicide may assist to reduce the probability of resistance. Considering their distinct mechanism of action, strobilurins are expected to have a high likelihood of developing resistance. Again, two to four applications of fungicide mixtures during the growing season increase yields by about two tonnes per hectare (Berry et al. 2008). The best method for managing leaf blotch disease is an integrated strategy that includes variety selection, cultural methods, crop rotation, and fungicides.

## 3. Conclusion

The current review suggests that leaf blotch disease is the correct disease name and it is caused by *Zymoseptoria tritici*. Moreover, leaf blotch (*Lb*) gene is the correct name of resistant gene than Septoria tritici blotch (*Stb*) gene. Different cultural, resistant wheat materials and biological methods of leaf blotch disease management have been advocated. Lastly, when we miss using the resistance materials and at high disease pressure, fungicide application at Gs 39 and after flowering is critical because the pathogen can harm the crop at this growth stage.

Pathogenicity levels, population diversity, and pathotype identification are also critical for investigating host resistance, but these are similarly restricted in some parts of the world. In developing countries, studies on epidemiological factors such as soil types, plant architecture, and other cultural practices are seriously lacking. Other work waiting for wheat pathologists includes molecular investigations on pathogen diversity and pathotype identification as well as spatial and temporal distribution study of the pathogen. Wheat durable and race-specific resistances have been weakened globally; as a result, regular breeding is crucial. Furthermore, it should be taken into account in future studies since prompt detection of virulent isolates is not always observed in certain countries. The probability of vertical resistance of wheat materials has been broken down, it may occur due to high genetic variability. Therefore, timely pathotype identification is very important. Different genes in horizontal and vertical resistance have been broken down; therefore, cloning or transferring resistant genes are very important. Gene pyramiding is very important to make the wheat gene diversities. Genomic regions of wheat cultivars need more attention to map the resistant gene areas on the chromosome.
